# Brain Connectometry Changes in Space Travelers After Long-Duration Spaceflight

**DOI:** 10.3389/fncir.2022.815838

**Published:** 2022-02-18

**Authors:** Andrei Doroshin, Steven Jillings, Ben Jeurissen, Elena Tomilovskaya, Ekaterina Pechenkova, Inna Nosikova, Alena Rumshiskaya, Liudmila Litvinova, Ilya Rukavishnikov, Chloë De Laet, Catho Schoenmaekers, Jan Sijbers, Steven Laureys, Victor Petrovichev, Angelique Van Ombergen, Jitka Annen, Stefan Sunaert, Paul M. Parizel, Valentin Sinitsyn, Peter zu Eulenburg, Karol Osipowicz, Floris L. Wuyts

**Affiliations:** ^1^Drexel University Neuroimaging Laboratory (DUN), Drexel University, Philadelphia, PA, United States; ^2^Lab for Equilibrium Investigations and Aerospace, University of Antwerp, Antwerp, Belgium; ^3^Imec-Vision Lab, University of Antwerp, Antwerp, Belgium; ^4^State Scientific Center of the Russian Federation – Institute for Biomedical Problem, Russian Academy of Sciences, Moscow, Russia; ^5^Laboratory for Cognitive Research, HSE University, Moscow, Russia; ^6^Radiology Department, National Medical Research Treatment and Rehabilitation Centre of the Ministry of Health of Russia, Moscow, Russia; ^7^Coma Science Group, GIGA Consciousness, University of Liège, Liège, Belgium; ^8^Department of Translational Neurosciences-ENT, University of Antwerp, Antwerp, Belgium; ^9^Department of Imaging & Pathology, Translational MRI, KU Leuven – University of Leuven, Leuven, Belgium; ^10^Department of Radiology, Royal Perth Hospital, University of Western Australia Medical School, Perth, WA, Australia; ^11^Faculty of Fundamental Medicine, Lomonosov Moscow State University, Moscow, Russia; ^12^Institute for Neuroradiology, Ludwig-Maximilians-University Munich, Munich, Germany

**Keywords:** spaceflight, magnetic resonance imaging (MRI), tractography, neuroplasticity, neuroscience, International Space Station (ISS), microgravity

## Abstract

Humans undergo extreme physiological changes when subjected to long periods of weightlessness, and as we continue to become a space-faring species, it is imperative that we fully understand the physiological changes that occur in the human body, including the brain. In this study, we present findings of brain structural changes associated with long-duration spaceflight based on diffusion magnetic resonance imaging (dMRI) data. Twelve cosmonauts who spent an average of six months aboard the International Space Station (ISS) were scanned in an MRI scanner pre-flight, ten days after flight, and at a follow-up time point seven months after flight. We performed differential tractography, a technique that confines white matter fiber tracking to voxels showing microstructural changes. We found significant microstructural changes in several large white matter tracts, such as the corpus callosum, arcuate fasciculus, corticospinal, corticostriatal, and cerebellar tracts. This is the first paper to use fiber tractography to investigate which specific tracts exhibit structural changes after long-duration spaceflight and may direct future research to investigate brain functional and behavioral changes associated with these white matter pathways.

## Introduction

The human brain retains a high degree of neuroplasticity into adulthood. Functionally dependent neuroplastic responses are maintained through the same mechanisms that allow for profound developmental and learning-dependent changes (Pascual-Leone et al., 2005). Additionally, the adult brain may be subject to mechanical forces that exert mass effects—changing the shape and microstructural organization of the brain (Laitinen et al., 2015). Spaceflight has the potential to profoundly alter both the function and shape of the adult brain. While the physiological effects of spaceflight have been studied for many decades, research into the effects of spaceflight on the brain is still in its infancy. The human desire to increase our exploration of space exacerbates the need to understand the effects of spaceflight on the human brain.

The first demonstration of brain anatomical changes after spaceflight entailed gray matter (GM) volume changes as measured by magnetic resonance imaging (MRI), which are suggestive of neuroplasticity and compression of the gyri (Koppelmans et al., 2016). Roberts et al. discovered a narrowing of the central sulcus, supravermian cistern, and calcarine sulcus on the post-flight MRI scans of their astronaut cohort (Roberts et al., 2017). They also saw an expansion of the brain ventricles and noticed an upward shift of the brain within the skull. Moreover, the changes reported in both studies appear to be specific to long-duration mission astronauts, which demonstrates the role of mission duration on these anatomical changes (Koppelmans et al., 2016; Roberts et al., 2017). We previously observed a widespread redistribution of CSF around the brain and dilated ventricles, as well as GM and white matter (WM) volume decreases (Van Ombergen et al., 2018). In a *post hoc* analysis, it was found that there was a mean increase in total ventricular volume of 11.6% (± 1.5%) post- compared to pre-flight (Van Ombergen et al., 2019). When contrasting follow-up (7 months post terrestrial return) versus preflight data, CSF volume shifts were still present, GM volume had only partially returned to preflight levels, and total ventricular volume remained increased by a mean of 6.4% (± 1.3%) (Van Ombergen et al., 2018, 2019). More recently, the post-flight increase in ventricular volume was shown to uphold even for a year after spaceflight (Kramer et al., 2020). These studies make it clear that the brain shifts superiorly and that intracranial GM, WM, and CSF exhibit volume changes as a result of spaceflight, with such effects at least partially persisting for a longer period of time. However, these studies are unable to elucidate whether these observations represent merely effects of mass movement or reshaping, effects of fluid redistribution, or whether there are any neuroplastic changes within the brain.

Utilizing diffusion MRI, it is possible to estimate the brain’s microstructure in each MRI voxel. It is also a technique that is sensitive to free-water changes, as is the case for the fluid shift effects of spaceflight. One study applied the diffusion tensor model to investigate spaceflight-induced changes in brain microstructure (Lee et al., 2019). Lee et al. (2019) found whole-brain shifts in CSF similar to what was observed in anatomical scans and, after correcting for each voxel’s free-water fraction, they demonstrated microstructural changes in the corticospinal tract, cerebellar pedunculi, as well as tracts connecting the occipital lobe with frontal and temporal lobes). However, one major limitation of the tensor model is that it cannot distinguish signals arising from multiple fiber populations that are present in one voxel. To overcome this limitation, the technique of spherical deconvolution was used in a recent study, where data collected from cosmonauts preflight, postflight, and at seven months follow up were analyzed (Jillings et al., 2020). This microstructural analysis technique revealed evidence of net increases in gray matter tissue in the basal ganglia and white matter tissue in the cerebellum, which provide strong signs of adaptive sensorimotor neuroplasticity (Jillings et al., 2020). Seven months after the space mission, these tissue increases were partially sustained, particularly noticeable in the cerebellum, although there was a large inter-individual variability. The researchers also demonstrated the previously reported fluid shift effects on the distribution of cerebral spinal fluid (CSF) and on the cortical morphology by investigating the relative compositions of the three main tissue types (GM, WM, and CSF) in each voxel in the brain. These mechanical, fluid shift effects mostly normalize at follow-up, with the exception of ventricular and ventral CSF expansions still being present.

In the present study, we aim to further our knowledge on structural changes in the brain after spaceflight at the level of deep-brain WM tracts. Specifically, we conducted a differential tractography analysis, meaning that tractography is performed along the voxels that exhibit changes in microstructural properties after spaceflight compared to before. This diffusion MRI technique is used for the first time in analyzing data of a population of space travelers. This approach enables us to validate previously reported brain microstructural changes after spaceflight and present these findings at the level of WM fiber tracts.

## Materials and Methods

### Study Design

Magnetic resonance imaging (MRI) scans of twelve male Roscosmos cosmonauts were acquired before and after their respective space flights between February 2014 and February 2020 at the National Medical Research Treatment and Rehabilitation Centre of the Ministry of Health of Russia. The cosmonauts all engaged in long-duration missions to the International Space Station (ISS) (mission length average: 172 days). Scans were acquired on average 89 days before spaceflight (preflight), a mean of ten days after return to Earth (Post Flight), and a final scan an average of 230 days after return from space (follow-up). All twelve cosmonauts were scanned pre and post-flight, while only eight cosmonauts opted in for the follow-up scan. In addition, thirteen age, gender, and education matched controls were scanned twice with similar intervals as the pre and post-flight scans of cosmonauts in order to account for changes due to aging. No follow-up data were available for the control group. All participants were tested for handedness using the Edinburgh Handedness Inventory (Oldfield, 1971), with a matched group of all right-handed controls and cosmonauts. An overview of cosmonaut and control group demographic information can be found in Table 1.

**TABLE 1:** Demographic information of cosmonaut and control group subjects.

	Cosmonauts *average (SD)*	Controls *average (SD)*	Two-sample *t*-test (p-value)
Age (years)	45 (5)	43 (6)	0.349
Mission duration (days)	172 (25)		
Previous mission experience (days)	199 (199)		
Preflight MRI – launch (days)	89 (34)		
Return – postflight MRI (days)	10 (3)		
Preflight MRI – postflight MRI (days)	270 (32)	240 (54)	0.099
Return – followup MRI (days)	230 (62)		

*Preflight and postflight MRI for the control group represents the two scanning sessions for this group. Statistical comparisons between the two groups were performed using a two-sample t-test (2-tailed). SD = standard deviation.*

The study was approved by the European Space Agency Medical Board, the Committee of Biomedicine Ethics of the Institute of Biomedical Problems of the Russian Academy of Sciences and the Human Research Multilateral Review Board. All participants were informed on the content and nature of the study and signed informed consent agreements.

### Magnetic Resonance Imaging Data Acquisition

A total of 12 preflight, 12 post-flight, and 8 follow-up diffusion MRI scans were acquired using a GE Discovery MR750 3T MRI system equipped with a 16-channel receiver head coil using a twice refocused pulsed gradient spin-echo echo-planar imaging sequence. An optimized multi-shell dMRI acquisition scheme was prescribed, containing diffusion weightings of b = 0, 700, 1200, and 2800 s/mm^2^, applied in 8, 25, 45, and 75 directions, respectively (Jeurissen et al., 2014). In addition, 3 b = 0 s/mm^2^ images were acquired with reversed-phase encoding, for the purpose of correcting susceptibility-induced distortions. Other imaging parameters were: repetition/echo time of 7800/100 ms, voxel size of 2.4 × 2.4 × 2.4 mm^3^, matrix size of 100 × 100, 58 slices, and 1 excitation. Imaging was accelerated by a factor of 2 using the Array coil Spatial Sensitivity Encoding Technique. The total acquisition time was 21 min and 23 s.

### Data Processing and Quality Control

All data were pre-processed according to the proposed pipeline by Cieslak et al. (2021). Steps included image denoising using Marcenko-Pastur principle components analysis [utilizing MRtrix’s dwidenoise (Veraart et al., 2016)], Eddy current distortions [utilizing FSL’s eddy (Andersson and Sotiropoulos, 2016)], susceptibility-induced distortion [utilizing FSL’s -topup (Andersson et al., 2003)] and bias field correction [utilizing ANTs’ N4BiasFieldCorrection (Tustison et al., 2010)].

To minimize the possibility of false positives, a stringent quality control process was applied [for details, see Yeh et al. (2019)]. First, image acquisition consistency between scans was confirmed; all scans were consistent across repetitions. Second, the correlation coefficient is calculated of neighboring low-b DWI volumes of similar gradient direction, with between-subject scan differences greater than r = 0.1 being rejected; this resulted in no subjects being excluded. Third, slice-wise signal dropout for each slice in each diffusion-weighted image was assessed, with an inclusion threshold of < 1% of slices being affected; all data sets passed this criterion. Fourth, b-table orientation was checked using the fiber coherence index (Schilling et al., 2019).

### Reconstruction

**FIGURE 1:**
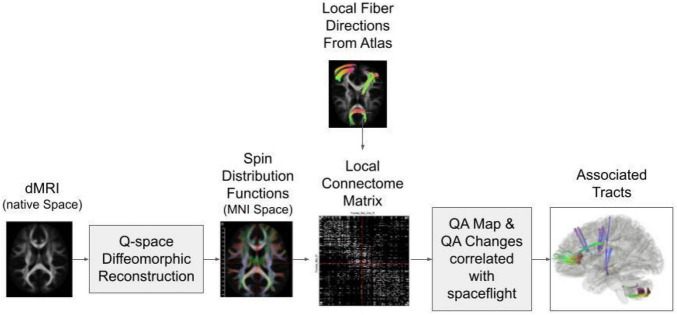
Diagram of the connectometry analysis process. The diffusion MRI (dMRI) data from all subjects are reconstructed in MNI space through Q-space diffeomorphic reconstruction. Then the spin distribution functions are calculated from the data and compared to the predicted local fiber directions from the atlas to create a connectome matrix. A quantitative anisotropy (QA) map is calculated based on the local connectome matrix. Tractography is performed along the voxels that show QA change correlations with spaceflight.

An overview of the reconstruction and tractography steps are illustrated in Figure 1. All scans were warped to the Montreal Neurological Institute (MNI) reference space based on the HCP-1021 young adult template using Q-Space diffeomorphic reconstruction (QSDR) (Yeh et al., 2018). The QSDR is an average of the q-sampling imaging which is used for the construction of the spin distribution functions in any given template space (e.g., MNI space) (Yeh et al., 2010). A quality check of the R-squared value is done to ensure no registration errors were made. The restricted diffusion was quantified using restricted diffusion imaging which uses Q-space imaging, a model-free approach that estimates the displacement distribution of diffusing spins. The displacement distribution derived from the q-space imaging is a three-dimensional probability density function of the diffusion displacement. The results are projected on a unit sphere to calculate its corresponding orientation distribution function (ODF). A diffusion sampling length ratio, which defines the radius of the diffusion spins included in the ODF estimation, of 1.25 was used (Yeh et al., 2017). The output resolution is 2 mm isotropic.

### Connectometry Analysis

The quantitative anisotropy (QA) was extracted as the local connectome fingerprint (LCF) (Yeh and Tseng, 2011) and used in the connectometry analysis. The LCF is DSI Studio’s way of sampling white matter characteristics as a “fingerprint.” The method uses QSDR to calculate the density of diffusing water along major fiber bundles from diffusion MRI. Diffusion MRI connectometry (Yeh et al., 2016) was used to derive the correlation tractography that has a longitudinal change of QA correlated with days in space for each cosmonaut. A T-score threshold of 1 was assigned and tracking was performed using a deterministic fiber tracking algorithm (Yeh et al., 2013) to obtain correlation tractography. Topology-informed pruning (Yeh et al., 2019) was conducted with 1 iteration to remove false connections. All tracks generated from bootstrap resampling were included. A length threshold of 20 mm distance was used to select tracks. The seeding number for each permutation was 50000. To estimate the false discovery rate (FDR), a total of 2000 randomized permutations were applied to the group label to obtain the null distribution of the track length. FDR analysis was used to determine the significance of findings for this research and was two-tailed due to the hypothesis not having directionality. The permutation (2000 permutations per analysis) was applied to subject labels to test results against the permuted condition. These null findings were then used to test the results under non-permutation conditions to compute the FDR automatically in DSI Studio. FDR can be interpreted as FDR ≤ 0.05 as being highly confirmatory, FDR = 0.05–0.2 as highly possible of positive findings, and FDR = 0.2–0.3 having a moderate possibility of positive findings. FDR > 0.3 shows non-significant findings (Yeh et al., 2019).

## Results

### Postflight Minus Preflight

**FIGURE 2:**
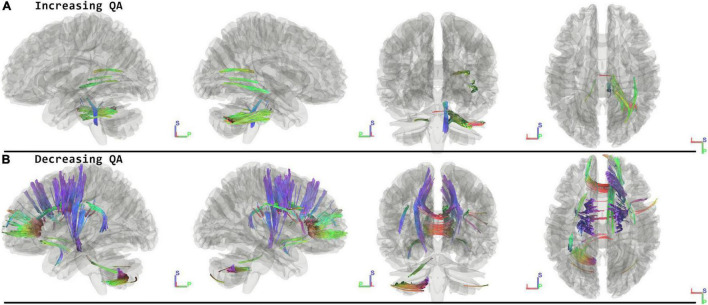
Tracts Associated with changes post minus preflight. Increasing quantitative anisotropy (QA) shows tracts increasing in the middle cerebellar peduncle, lemniscus, and corpus callosum (FDR = 0.0033) **(A)**. Decreasing QA shows changes in the frontal lobes, corpus callosum, and cerebellum (FDR = 0.0009) **(B)**. Blue indicates superior – inferior. Green indicates anterior – posterior. Red indicates left – right.

When running the contrast of postflight minus preflight, there were significant results for increasing QA (FDR = 0.0033) located in the Middle Cerebellar Peduncle, Right Medial Lemniscus, Corpus Callosum Forceps Major, and Right Inferior Fronto Occipital Fasciculus. For decreasing QA there were significant tracts in the Corpus Callosum Forceps Minor, Right Dentatorubrothalamic Tract, Vermis, Corpus Callosum Body, Left Corticospinal Tract, Right Corticostriatal Tract Anterior, Left Corticopontine Tract Parietal, Right Corticostriatal Tract Superior, Left Arcuate Fasciculus, and Left Corticostriatal Tract Superior (FDR = 0.0009) (Figure 2 and Table 2).

**TABLE 2:** Overview of the tract labels that show changes in quantitative anisotropy (QA) for the different contrasts used in this analysis.

Contrast	Tract (Atlas: HCP1021) (All data is presented in neuroanatomical convention- Left on Left)	Direction and FDR	QA (Standard Error)
Postflight – Preflight	• Corpus Callosum Forceps Major • Inferior Fronto Occipital Fasciculus, R • Medial Lemniscus, R • Middle Cerebellar Peduncle, Bilateral	**INCREASING QA** (FDR = 0.0033)	**0.28 (± 0.015)**
Postflight – Preflight	• Arcuate Fasciculus, L • Corpus Callosum Body • Corpus Callosum Forceps Minor • Corticopontine Tract Parietal, L • Corticospinal Tract, L • Corticostriatal Tract Anterior, R • Corticostriatal Tract Superior, R • Corticostriatal Tract Superior, L • Dentatorubrothalamic Tract, R • Vermis	**DECREASING QA** (FDR = 0.0009).	**0.26 (± 0. 013)**
Followup – Postlight	• Anterior Commissure • Cingulum Frontal Parietal, R • Corpus Callosum Body • Corpus Callosum Forceps Minor • Corticostriatal Tract Anterior, R • Corticostriatal Tract Anterior, L • Middle Cerebellar Peduncle Bilateral • Uncinate Fasciculus, L	**INCREASING QA** (FDR = 0.0567).	**0.27 (± 0.014)**
Followup – Postlight	• Cingulum Parahippocampal Parietal, R • Corpus Callosum Body • Corpus Callosum Forceps Major • Corpus Callosum Tapetum • Corticostriatal Tract Posterior, R • Corticostriatal Tract Posterior, L • Inferior Longitudinal Fasciculus, R • Thalamic Radiation Posterior, R • Thalamic Radiation Posterior, L	**DECREASING QA** (FDR = 0.0014)	**0.43 (± 0.022)**
Followup – Preflight	The connectometry analysis found no significant result in tracks with increased QA.	**INCREASING QA** (FDR = 1.00)	**N/A**
Followup – Preflight	• Arcuate Fasciculus, L • Cerebellum, R • Cerebellum, L • Corpus Callosum Body • Corpus Callosum Forceps Major • Corpus Callosum Forceps Minor • Corpus Callosum Tapetum • Corticopontine Tract Parietal, L • Corticospinal Tract, L • Corticostriatal Tract Posterior, L • Medial Lemniscus, L • Middle Cerebellar Peduncle Bilateral • Superior Longitudinal Fasciculus, R • Vermis	**DECREASING QA** (FDR = 0.0069)	**0.36 (± 0.018)**

*The right column shows the average QA change for all significant tracts of the associated contrast. N/A is used for contrasts where no significant tracts show QA changes. R = right, L = left.*

### Follow-Up Minus Postflight

**FIGURE 3:**
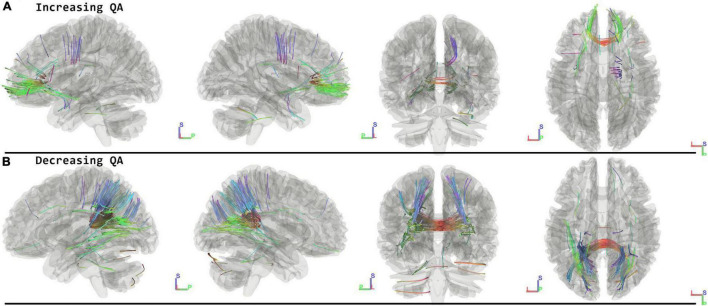
Tracts Associated with changes of followup minus postflight. Increasing QA shows changes in forceps minor, corpus callosum, corticostriatal, and right cingulum (FDR = 0.0567) **(A)**. Decreasing QA shows changes in the forceps major, corpus callosum, and parietal lobe (FDR = 0.0014) **(B)**. Blue indicates superior – inferior. Green indicates anterior – posterior. Red indicates left – right.

When running the contrast of follow-up minus postflight, there were significant results for increasing QA (FDR = 0.0567) located in the Corpus Callosum Forceps Minor, Left Uncinate Fasciculus, Corpus Callosum Body, Right Corticostriatal Tract Anterior, Anterior Commissure, Left Corticostriatal Tract Anterior, Middle Cerebellar Peduncle, Right Cingulum Frontal Parietal. For decreasing QA there were changes located in the Corpus Callosum Forceps Major, Left Thalamic Radiation Posterior, Corpus Callosum Tapetum, Corpus Callosum Body, Left Corticostriatal Tract Posterior, Right Inferior Longitudinal Fasciculus, Right Thalamic Radiation Posterior, Right Cingulum Parahippocampal Parietal, Right Corticostriatal Tract Posterior showing decreased QA (FDR = 0.0014) (Figure 3 and Table 2).

### Follow-Up Minus Preflight

**FIGURE 4:**
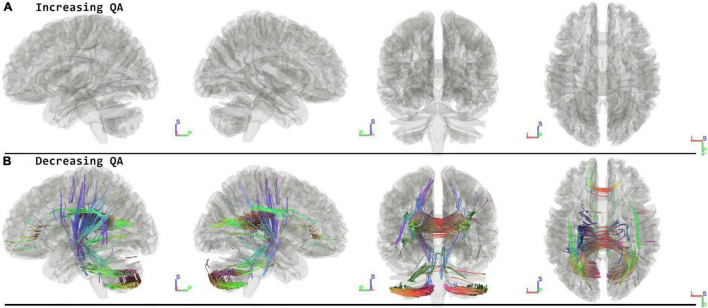
Tracts associated with changes of followup minus preflight. There are no significant changes in increasing QA **(A)**. For decreasing QA there were changes in the corpus callosum, cerebellum, lemniscus, corticopontine tract, and corticostriatal tract (FDR = 0.0069) **(B)**. Blue indicates superior – inferior. Green indicates anterior – posterior. Red indicates left – right.

When running the contrast of follow-up minus preflight, there were no significant results for increasing QA (FDR = 1.00). There were significant results for decreasing QA in the Corpus Callosum Forceps Major, Corpus Callosum Tapetum, Vermis, Right Cerebellum, Left Corticopontine Tract Parietal, Left Cerebellum, Left Medial Lemniscus, Middle Cerebellar Peduncle, Left Corticostriatal Tract Posterior, Corpus Callosum Body, Left Corticospinal Tract, Right Superior Longitudinal Fasciculus, Corpus Callosum Forceps Minor, Left Arcuate Fasciculus (FDR = 0.0069) (Figure 4 and Table 2).

### Control Group

Thirteen age, gender, and handedness matched controls were included and scanned twice on the same MRI scanner as the cosmonauts with an interval of 240 days in between. The controls were analyzed using the same methods as the experimental group and there were no significant findings (FDR = 1.00).

## Discussion

Our findings demonstrate widespread microstructural changes associated with long-duration space flight within the sensorimotor tracts (e.g., corticopontine, corticospinal, and corticostriatal tracts) including many tracts connecting the cerebellum (dentatorubrothalamic tract, middle cerebellar peduncle, vermis), as well as within the corpus callosum, inferior fronto occipital fasciculus (IFOF) and arcuate fasciculus. Several of these tracts exhibit opposite microstructural changes during the seven-month period after the space mission, including the corpus callosum and corticostriatal tracts, suggesting normalization to preflight levels. However, compared to preflight, the seven-month follow-up measurement reveals that many microstructural changes associated with long-duration spaceflight remain present. No changes were detected in a control group scanned twice over a similar time interval as the duration of the space mission, thereby excluding effects of aging. These microstructural changes may reflect different sources of effects of spaceflight on the brain, including fluid shift effects, brain anatomical shifts, and neuroplasticity.

### Immediate Effects—Postflight Minus Preflight

Consistent with the effects previously observed with diffusion MRI (Jillings et al., 2020), we confirm changes in multiple tracts associated with motor and sensory functions, including the left corticospinal tract (Thomas and Gorassini, 2005), portions of the corticostriatal tracts and right dentatorubrothalamic tract (Petersen et al., 2018). Considering the different physics and kinesthetics applying to the extreme environment of space and the hypothesis that these have significant effects on the brain’s representation and control of the body, these tracts are therefore suspected to reflect this altered sensorimotor function shown in space travelers (Reschke et al., 1998). While brain anatomical shape changes and fluid redistribution can’t be excluded as potential driving factors for our observations, it is worth noting that in the same sample of cosmonauts the primary sensorimotor cortex (M1/S1), the basal ganglia, and the cerebellum showed microstructural changes with no change in free-water fraction and which accounted for local deformations (Jillings et al., 2020). Structural changes in the cerebellum have also been observed in another diffusion MRI study in space crew (Lee et al., 2019), highlighting that various techniques applied in different space crews all reveal similar effects in the cerebellum. This demonstrates the importance of additional work to fully unravel the exact nature of spaceflight’s effect on the cerebellum and its functional relevance.

We also found the corpus callosum to show widespread structural changes across several of its subdivisions for nearly all time point comparisons in this analysis and for different directions of QA change. These findings are more likely explained by brain anatomical shifts than neuroplasticity or parenchymal fluid shifts. Previous work has characterized a number of specific anatomical shifts, including ventricular enlargement, uplifting of the brain, narrowing of the longitudinal fissure, and displacements of deep-brain structures (Roberts et al., 2017; Van Ombergen et al., 2018, 2019). Considering that the corpus callosum is less bulky compared to most other parts of the brain, it may be more susceptible to strain caused by this assembly of anatomical changes. In addition, the arcuate fasciculus might also be specifically more prone to anatomical brain shifts because of the bent shape of this tract. The arcuate fasciculus connects temporal with frontal lobes through a U-shaped tract, and considering that the Sylvian fissure is widened after spaceflight (Van Ombergen et al., 2018; Jillings et al., 2020), the relative displacements of the temporal and frontal lobes may therefore affect the anatomical structure of the arcuate fasciculus, leading to the observed changes in this tract.

From a functional perspective, the forceps minor mediates the connectivity of the inferior-lateral and orbital parts of the frontal lobes, regions with known roles in social cognition (McDonald et al., 2019), executive function (Mamiya et al., 2018), and also gait function (Seiler et al., 2017). The arcuate fasciculus connects temporal with inferior frontal lobes for important roles in language. The functional descriptions and anatomical connections of these tracts that show changes after spaceflight may trigger future work to investigate behavioral changes possibly associated with structural reorganization through appropriate neuropsychological tests or alternative neuroimaging analyses.

### Normalization Effects—Follow Up Minus Postflight

The results at follow-up demonstrate the occurrence of opposite effects with respect to the changes observed from pre- to post-flight in several tracts. Specifically, the corticostriatal tracts and frontal crossing fibers (forceps minor and corpus callosum body) show QA decreases from pre- to post-flight and increases from post-flight to follow-up. The posterior crossing fibers (forceps major) exhibit the opposite direction of effects for the two contrasts. Based on previous reports of long-term follow-up data, we know that the morphological changes at the superior surface of the brain after spaceflight, which together demonstrate the upward brain shift, appear to reverse back to the preflight state (Van Ombergen et al., 2018; Jillings et al., 2020). Also, the structural changes in the basal ganglia and sensorimotor cortex that can be explained by neuroplasticity as previously reported (Jillings et al., 2020) showed largely reversed effects during the follow-up period of seven months on earth.

Several tracts show significant changes between post-flight and follow-up, while they did not show significant effects after spaceflight compared to before. These include the cingulum, the inferior longitudinal fasciculus (ILF), and the posterior thalamic radiations. The ILF connects the occipital and temporal-occipital areas of the brain and is related to processing and modulating visual cues (Herbet et al., 2018), the posterior thalamic radiations have been shown to be linked to visual short-term memory (Menegaux et al., 2017), and the cingulum can be considered a part of the limbic system and may have a role in interoception and emotional processing (Nakajima et al., 2019).

### Residual Effects—Follow Up Minus Preflight

Previous work on structural changes after spaceflight that included a follow-up measurement over six months post-flight revealed remaining effects of the space mission on the brains of cosmonauts (Van Ombergen et al., 2018, 2019; Jillings et al., 2020). These include ventricular enlargement, Sylvian fissure expansion, and subarachnoid CSF enlargement along the ventral side of the brain. One study also found widespread GM and WM volume changes occurring during this post-flight follow-up period (Van Ombergen et al., 2018). Based on these results, there seems to be a slow normalization phase with so far a lack of data on when or whether a complete pre-flight state is reached. In the current study, we observe longer-term effects in the corpus callosum and arcuate fasciculus, which might be explained by the remaining expansion of the ventricles and Sylvian fissure. On the other hand, sensorimotor and cerebellar tracts also exhibit remaining effects of spaceflight. One previous study that included these follow-up measurements also revealed some remaining changes in the cerebellum, although they were highly variable across subjects and spatially encompassed a smaller area (Jillings et al., 2020). It is likely that some of the cerebellar tracts shown in the current study are remaining effects of spaceflight due to anatomical shifts.

From a general point of view, the current observations fall under similar trends reported by previous neuroimaging work in space crew, namely widespread structural changes caused by long-duration spaceflight that partially reverse to pre-flight levels in the longer term (Van Ombergen et al., 2018, 2019; Jillings et al., 2020; Kramer et al., 2020), as well as effects that are specific to the follow-up period after the space mission (Van Ombergen et al., 2018). Understanding the evolution in time of these structural changes remains an important focus for future research (Roberts et al., 2020).

### Limitations

This study had a few limitations with the largest being the size of the sample. Due to the extreme nature of our population being space travelers and the fact this is per definition a small cohort, we were limited with our sample. Furthermore, the follow-up time point only saw eight participating cosmonauts. However, there remains very few reports on this type of data so far. The rarity of subjects who have spent extensive time in space marks the importance of extracting as much information as we can from every subject. Another limitation is that the sample contains cosmonauts who flew previous missions prior to the collection of the baseline (preflight) data. This may alter the baseline data due to persistent effects from the previous missions. This limitation, however, is partially compensated by performing longitudinal paired analyses. Another limitation is that the post-flight time point was acquired an average of ten days after return from space, thus resulting in a possible underestimation in the effects measured. One limitation with respect to the adopted diffusion MRI technique is that we cannot disentangle effects that result from fluid shifts, brain anatomical shape changes, or neuroplasticity. However, for the purpose of this work, we aimed to characterize the full scope of detectable structural changes at the level of WM tracts to potentially drive hypotheses for future analyses in space travelers. Future research may overcome some of these limitations by employing complementary neuroimaging techniques, such as functional connectivity MRI, which would augment our understanding of the anatomical changes by exploring changes in functional connectivity, further disambiguating fluid shifts from neuroplastic responses. Lastly, without access to appropriate measures of neuropsychological performance, it is impossible to fully understand the functional associations of the effects reported here. Further behavioral testing will be necessary to understand the effects of spaceflight on the brain.

### Future Implications

These findings suggest that just as understanding the effects of pressure and oxygen deficiency on the human body was imperative for flight within the atmosphere, a similar understanding of how spaceflight affects our body is necessary for the exploration of the final frontier. As missions to Mars are at a minimum of nine months of spaceflight, more long-term studies on the human brain will need to be conducted. Current countermeasures exist for muscle and bone loss (Stein, 2013), but if future research provides evidence that countermeasures are necessary for the brain, then we must begin to answer this challenging question. Many researchers and thinkers (T͡Siolkovskiǐ and Syers, 1960) have asked the question of how to simulate the earth-level effects of gravity in space and no single proposal has yet solved the issue. With the use of the centripetal force to create a rotating spaceship comes two issues: the first being that a very large spacecraft of at least 15 m wide would be necessary in order to achieve the rotation and radius necessary for any meaningful gravity (Clément et al., 2015), and the second being that even in a large rotating cylinder the uneven distribution of forces would impact different parts of a person’s body. This effect would translate to a person feeling more G forces at their feet than their head. The Nautilus-X was a project proposed for the ISS in 2011 by NASA as a sleep pod that would give 0.11–0.51 g while astronauts would sleep, but would likely experience vestibular confusion due to the changes in weightlessness between sleep and wake (Holderman and Henderson, 2011). Other answers for artificial gravity may exist in the creation of gravity as scientists funded by ESA have created a device that has shown to create gravitomagnetism, although it produced only 0.0001g (Tajmar et al., 2006). The future of artificial gravity may be very different than what we currently envision. It is imperative that space agencies look toward new methods of achieving artificial gravity in order to extend the distance and duration that humans can travel in space.

## Conclusion

Our study extends on the known effects of long-duration spaceflight on brain structure by determining which exact WM bundles underlie these changes. We found structural changes after spaceflight compared to before in tracts that from a functional perspective have roles in sensorimotor, language and visual function, and from an anatomical perspective may be susceptible to morphological changes due to brain and intracranial fluid shifts. However, the neuropsychological data to analyze the functional correlates of any observed changes is not available, underlying the importance of a complete neuropsychological profile of these high function individuals. Changes occurring between the early post-flight and a seven-month follow-up period indicate some normalization, while also differences in WM structure between the pre-flight and follow-up time points remain. This work therefore demonstrates structural changes in specific white matter tracts as a result of long-duration spaceflight, which may form a basis for future research into the full scope of brain changes in preparation for human exploration of the final frontier.

## Data Availability Statement

The original contributions presented in the study are included in the article/Supplementary Material, further inquiries can be directed to the corresponding author.

## Ethics Statement

The studies involving human participants were reviewed and approved by European Space Agency Medical Board, the Committee of Biomedicine Ethics of the Institute of Biomedical Problems of the Russian Academy of Sciences and the Human Research Multilateral Review Board. The patients/participants provided their written informed consent to participate in this study.

## Author Contributions

ET, SS, PP, SL, JS, FW, and BJ contributed to the conception of the work. ET, EP, IR, SS, PP, VS, SL, JS, FW, and BJ contributed to the design of the work. SJ, AV, AR, LL, IN, EP, IR, CS, CD, VS, VP, BJ, and FW contributed to the data acquisition. AD and KO contributed to data analysis. AD, KO, SJ, EP, PE, FW, and BJ contributed to data interpretation. AD, SJ, and KO contributed to drafting the manuscript. BJ, EP, JA, and FW contributed to a substantial revision of the manuscript. All authors contributed to the article and approved the submitted version.

## Conflict of Interest

The authors declare that the research was conducted in the absence of any commercial or financial relationships that could be construed as a potential conflict of interest.

## Publisher’s Note

All claims expressed in this article are solely those of the authors and do not necessarily represent those of their affiliated organizations, or those of the publisher, the editors and the reviewers. Any product that may be evaluated in this article, or claim that may be made by its manufacturer, is not guaranteed or endorsed by the publisher.
